# Twinning in metastable high-entropy alloys

**DOI:** 10.1038/s41467-018-04780-x

**Published:** 2018-06-18

**Authors:** Shuo Huang, He Huang, Wei Li, Dongyoo Kim, Song Lu, Xiaoqing Li, Erik Holmström, Se Kyun Kwon, Levente Vitos

**Affiliations:** 1 Applied Materials Physics, Department of Materials Science and Engineering, Royal Institute of Technology, Stockholm, SE-100 44 Sweden; 2 Science and Technology on Surface Physics and Chemistry Laboratory, Mianyang, 621900 PR China; 3 Department of Physics and Astronomy, Division of Materials Theory, Uppsala University, SE-75120 Uppsala, Sweden; 4 Department of Physics, Pukyung National University, Busan, 608-737 Republic of Korea; 5 Sandvik Coromant R&D, 126 80 Stockholm, Sweden; 6 Graduate Institute of Ferrous Technology, Pohang University of Science and Technology, Pohang, 37673 Republic of Korea; 7 Institute for Solid State Physics and Optics, Wigner Research Centre for Physics, H-1525 Budapest, Hungary

## Abstract

Twinning is a fundamental mechanism behind the simultaneous increase of strength and ductility in medium- and high-entropy alloys, but its operation is not yet well understood, which limits their exploitation. Since many high-entropy alloys showing outstanding mechanical properties are actually thermodynamically unstable at ambient and cryogenic conditions, the observed twinning challenges the existing phenomenological and theoretical plasticity models. Here, we adopt a transparent approach based on effective energy barriers in combination with first-principle calculations to shed light on the origin of twinning in high-entropy alloys. We demonstrate that twinning can be the primary deformation mode in metastable face-centered cubic alloys with a fraction that surpasses the previously established upper limit. The present advance in plasticity of metals opens opportunities for tailoring the mechanical response in engineering materials by optimizing metastable twinning in high-entropy alloys.

## Abstract (web summary)

Twinning has been experimentally seen in high-entropy alloys, but understanding how it operates remains a challenge. Here, the authors show that twinning can be a primary deformation mechanism in three well-known medium- and high-entropy alloys that have unstable face-centered cubic lattices.

## Introduction

Designing strong, and at the same time, ductile metals has been among the most ambitious goals for metallurgists. The apparently conflicting mechanisms often resulted in grades compromising between strength and ductility. In spite of the early ideas revolving around Fe–Mn alloys^1^, simultaneous improvement of the two properties has been very challenging. The discovery of the advanced high-strength steels near the turn of the century was a pioneering step towards reaching such goals^2^. Recently, the strength–ductility tradeoff was also overcome by designing dual-phase high-entropy alloys (HEAs)^3^, which turned attention towards non-conventional alloys containing multi-principal elements in equal or nearly equal concentrations^4,5^. Successful single-phase solutions also exist such as the fracture-resistant HEAs^6^ and the exceptional damage-tolerance medium-entropy alloys (MEAs)^7^. Further, HEAs with surprising and unanticipated properties are being continuously put forward^8–18^. Parallel with the experimental efforts, computational modeling was also advanced to optimize compositions and reveal the atomic-level mechanisms^19^ behind the strength and ductility improvements.

The common denominator of the most promising routes over the strength–ductility dilemma is the tailored plastic deformation mechanism. Face-centered cubic (fcc) metals normally deform by dislocation glide resulting in good ductility and reduced strength. But in the aforementioned special alloys, the leading deformation modes are twinning and transformation induced plasticity. In particular, twinning was found to play an important role for the enhanced ductility and high strain hardening rate^2,3,6,7,20,21^. There are several phenomenological plasticity models^22,23^ developed to understand the activation of twinning deformation in fcc metals. These models are based on the stacking fault energy (SFE) defined as the excess energy due to the stacking fault connecting the Shockley partial dislocations. For instance, austenitic steels with SFE higher than 10–16 mJ m^−2^ are predicted to show twinning^24–26^, whereas alloys with SFE below this critical value should exhibit deformation-induced phase transformation. Theoretical works also addressed the question of twinnability^27–29^, and they indicate twinning for fcc stable metals with twinning fraction below 50%^27^.

Within the simplest model, the SFE corresponds to the energy difference between the hexagonal close-packed (hcp) and fcc structures^30^. Thus, the sizable positive SFE required for twinning by the phenomenological models^22,23^ implies a thermodynamically stable fcc phase. Traditional alloys like austenitic steels^31^ belong to this group. Since the discovery of the first fcc HEAs based on 3d transition metals^4,5^, it was expected that these multi-component systems also follow a similar plastic deformation scenario. However, recent experimental works reported a pressure-induced fcc–hcp transition in CrMnFeCoNi, and the hcp phase was retained after decompression to ambient pressure^32,33^. The observed equilibrium phase boundary between the fcc and hcp phases was placed near 600 K^33^. For the multi-component alloys in question^3,6,7,20,34–37^, including CrMnFeCoNi, ab initio theory fully confirms the experimental findings by predicting a thermodynamically unstable fcc lattice at low temperature. Hence, both experiment and theory suggest that the metastable fcc CrMnFeCoNi alloy should possess very small positive or negative SFEs at ambient and cryogenic conditions. But such SFE values in connection with the phenomenological models^22,23^ clearly fail to explain the observed twinning^6^. Thus, the available experimental and theoretical evidences severely challenge the validity of the widely-used SFE-based plasticity models in the case of metastable HEAs^22,27^. Although this fundamental problem was recognized by several groups^19,34–38^, so far no unified solution has been put forward.

Here, we employ first-principle quantum mechanical tools to resolve the twinning mechanism in metastable multi-component alloys. We select the representative Fe_80−*x*_Mn_*x*_Co_10_Cr_10_ (30 ≤ *x* ≤ 45) and CrMnFeCoNi HEAs^3,6^, and CrCoNi MEA^7^, for which reliable experimental data for the deformation mechanisms exist. Based on the intrinsic energy barriers, we show that it is the particular chemistry that is primarily responsible for the emergence of twinning in metastable alloys, and decreasing temperature efficiently drives the system across various deformation modes. The inherent affine shear strain prior to slip, which is associated to the relatively large critical twinning stress characteristic to the present systems, further promotes twinning. Our results are expected to help optimizing the properties of engineering materials involving HEAs towards excellent strength–ductility combinations.

## Results

### Deformation modes

The leading deformation modes in fcc metals are stacking fault (SF), twinning (TW), and full-slip (SL). The active mode is decided by the competition between the corresponding effective energy barriers (EEBs): $\bar \gamma _{{\mathrm{SF}}}\left( \theta \right) = \gamma _{{\mathrm{usf}}}/\cos \theta$, $\bar \gamma _{{\mathrm{TW}}}\left( \theta \right) = (\gamma _{{\mathrm{utf}}} - \gamma _{{\mathrm{isf}}})/\cos \theta$, and $\bar \gamma _{{\mathrm{SL}}}\left( \theta \right) = (\gamma _{{\mathrm{usf}}} - \gamma _{{\mathrm{isf}}})/\cos (60^\circ - \theta )$ for SF, TW, and SL, respectively^27^. In these expressions, *θ* varies between 0° and 60° and reflects the effect of grain orientation relative to the resolved shear direction, $\gamma _{{\mathrm{isf}}}$ is the intrinsic SFE, $\gamma _{{\mathrm{usf}}}$ the unstable SFE, and $\gamma _{{\mathrm{utf}}}$ the unstable twin fault energy. These intrinsic material parameters (IMPs) describe the generalized SFE (*γ*-surface)^39^ and are determined from a series of ab initio calculations. According to the above EEBs, twinning is possible for $\bar \gamma _{{\mathrm{TW}}}\left( \theta \right) \le \bar \gamma _{{\mathrm{SF}}}\left( \theta \right)$, otherwise martensitic transformation occurs. Furthermore, due to the shear direction dependence, SF and SL or TW and SL can co-exist in a homogenous material, but SF and TW are exclusive to each other independently of *θ* (they possess similar Schmid factors). We mention that SF and TW can coexist in special alloys (e.g., interstitial HEAs^16–18^) due to the broadening of their boundary. For the sake of simplicity, in the following we do not always mention SL when discussing the SF and TW mechanisms.

**Fig. 1:**
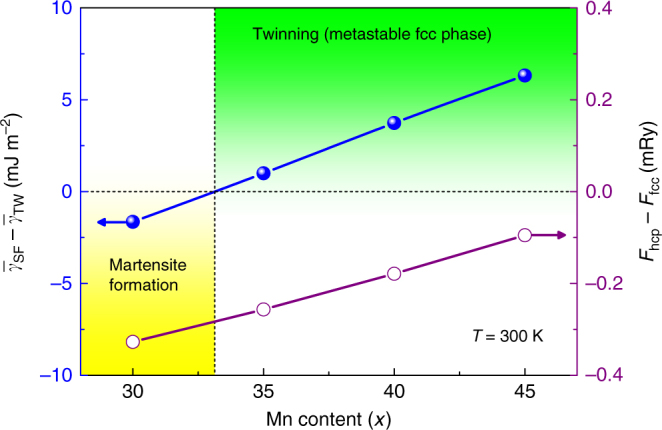
Composition-dependent effective energy barrier difference $\left( {\bar \gamma _{{\mathrm{SF}}} - \bar \gamma _{{\mathrm{TW}}}} \right)$ for the Fe_80−*x*_Mn_*x*_Co_10_Cr_10_ alloys at room-temperature. Negative $\left( {\bar \gamma _{{\mathrm{SF}}} - \bar \gamma _{{\mathrm{TW}}}} \right)$ indicates hcp martensite formation (yellow area), while positive one implies twinning (green area). Open circles denote the structural energy difference ($F_{{\mathrm{hcp}}} - F_{{\mathrm{fcc}}}$), where $F_{{\mathrm{hcp}}}$ and $F_{{\mathrm{fcc}}}$ are the free energies of hcp and fcc structures, respectively

Using the calculated EEBs, first we explore the deformation modes of the Fe_80−*x*_Mn_*x*_Co_10_Cr_10_ alloys as a function of temperature and composition. These alloys are reported to exhibit twinning or martensitic transformation depending on Mn content^3^. The room-temperature $\left( {\bar \gamma _{{\mathrm{SF}}} - \bar \gamma _{{\mathrm{TW}}}} \right) \equiv$ $\bar \gamma _{{\mathrm{SF}}}\left( {0^\circ } \right) - \bar \gamma _{{\mathrm{TW}}}\left( {0^\circ } \right)$ values are plotted in Fig. 1 as a function of Mn content. We find that $\left( {\bar \gamma _{{\mathrm{SF}}} - \bar \gamma _{{\mathrm{TW}}}} \right)$ changes sign with increasing Mn concentration. Namely, for *x* ≥ 33, TW is favored against SF, whereas for *x* < 33 SF is activated. In addition, SL is present in both regimes with *θ* increasing towards 60° (not shown). These theoretical predictions are in perfect agreement with observations^3^ and confirm that the present theory correctly captures the observed trends of the deformation modes as a function of composition.

Figure 1 also shows the free energy difference $\Delta F \equiv (F_{{\mathrm{hcp}}} - F_{{\mathrm{fcc}}})$ for Fe_80−*x*_Mn_*x*_Co_10_Cr_10_ by varying *x*. Negative Δ*F* indicates a thermodynamically unstable fcc lattice, which turns out to be the case for all compositions considered here. By combining the behavior of Δ*F* with that of $\left( {\bar \gamma _{{\mathrm{SF}}} - \bar \gamma _{{\mathrm{TW}}}} \right)$ in terms of Mn content, we arrive to a very interesting and unprecedented phenomenon. Namely, in Fe_80−*x*_Mn_*x*_Co_10_Cr_10_ HEAs, the room-temperature twinning is associated with a metastable fcc phase for 33 ≤ *x* ≤ 50 (upper limit is estimated by extrapolating the slope of Δ*F* towards high Mn content). We emphasize that since these alloys possess negative or very small positive $\gamma _{{\mathrm{isf}}}$ (as shown below), the empirical plasticity models based on SFE^22,23^ would dictate SF rather than TW mechanism, opposed to the present theoretical and former experimental findings^3^. Actually, in these special alloys, $\bar \gamma _{{\mathrm{TW}}}$ is lower than $\bar \gamma _{{\mathrm{SF}}}$, which induces twins instead of stacking faults toward martensitic transformation, despite of the negative Δ*F*. Below, we refer to this phenomenon as metastable twinning (MTW).

**Fig. 2:**
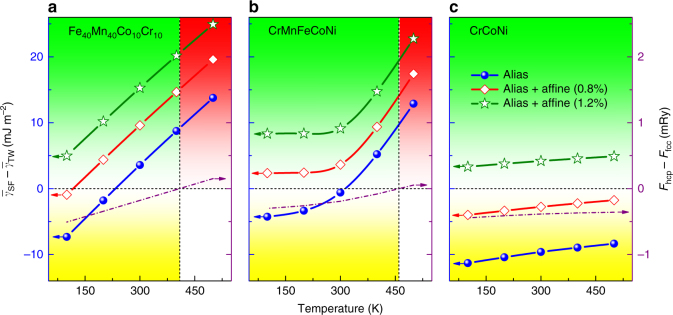
Temperature-dependent effective energy barrier difference $\left( {\bar \gamma _{{\mathrm{SF}}} - \bar \gamma _{{\mathrm{TW}}}} \right)$. **a**–**c** show the results for Fe_40_Mn_40_Co_10_Cr_10_, CrMnFeCoNi, and CrCoNi alloys respectively. The structural energy differences ($F_{{\mathrm{hcp}}} - F_{{\mathrm{fcc}}}$) are plotted by dash-dot lines for comparison. Solid lines with symbols denote $\left( {\bar \gamma _{{\mathrm{SF}}} - \bar \gamma _{{\mathrm{TW}}}} \right)$ without (alias, blue filled symbols) and with 0.8% and 1.2% affine shear strains (open symbols), respectively. The metastable twinning regime (MTW) is marked by the green, the martensite formation regime (SF) by the yellow, and the normal twinning regime (TW) by the red areas

Next, we consider the effect of temperature on the deformation modes. We recall that tuning the deformation mechanisms by temperature is a well-known routine to alter the plasticity of materials^6,7^. Figure 2a (solid symbols) displays $\left( {\bar \gamma _{{\mathrm{SF}}} - \bar \gamma _{{\mathrm{TW}}}} \right)$ and Δ*F* as a function of temperature for the Fe_40_Mn_40_Co_10_Cr_10_ HEA. Alloys with different Mn-levels follow qualitatively the same behavior and thus they are not included in the figure. At high temperature, the system is in the positive Δ*F* and TW + SL regime. Similar normal behavior is observed at very low temperature, where negative Δ*F* is associated with SF + SL. However, there exists a temperature interval where the Fe_80−*x*_Mn_*x*_Co_10_Cr_10_ HEAs behave unexpectedly: TW is activated in spite of the negative Δ*F*. For *x* = 35, 40, and 45, the abnormal interval includes room-temperature, whereas for *x* = 30 it is shifted above room-temperature. Similar calculated $\left( {\bar \gamma _{{\mathrm{SF}}} - \bar \gamma _{{\mathrm{TW}}}} \right)$ and Δ*F* versus temperature data for CrMnFeCoNi and CrCoNi alloys are shown in Fig. 2b,c (solid symbols), respectively. According to the free energy differences, for these two alloys, the hcp structure is more stable than the fcc one at low temperatures. Our findings regarding the structural stability are fully consistent with the previous theoretical works^34–36,40,41^ (see also Supplementary Note 1 and Supplementary Figs. 1 and 2). When comparing the trends for $\left( {\bar \gamma _{{\mathrm{SF}}} - \bar \gamma _{{\mathrm{TW}}}} \right)$ and Δ*F*, it turns out that the peculiar MTW appears in the CrMnFeCoNi alloy between ~300 and ~460 K, and the metastable CrCoNi alloy shows no twinning in the temperature range considered here. Before contrasting these theoretical predictions with the experimental observations, we investigate the calculated generalized SFEs.

### Generalized stacking fault energy

**Fig. 3:**
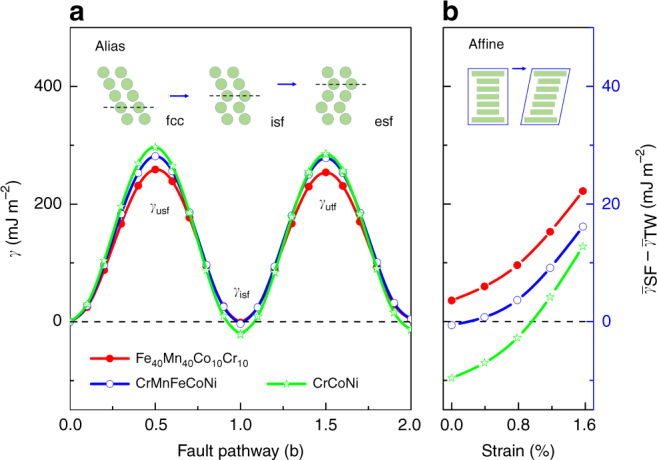
Generalized stacking fault energy curves for the Fe_40_Mn_40_Co_10_Cr_10_, CrMnFeCoNi, and CrCoNi alloys at room-temperature. In panel **a**, ${\bf{b}} = \frac{1}{6}\langle 11\bar 2\rangle$ is the Burgers vector of the partial dislocation (in units of fcc lattice parameter). $\gamma _{{\mathrm{isf}}}$, intrinsic stacking fault energy; $\gamma _{{\mathrm{usf}}}$, unstable stacking fault energy; $\gamma _{{\mathrm{utf}}}$, unstable twining fault energy. Panel **b** shows $\left( {\bar \gamma _{{\mathrm{SF}}} - \bar \gamma _{{\mathrm{TW}}}} \right)$ as a function of the affine shear strain

The room-temperature *γ*-surfaces of Fe_40_Mn_40_Co_10_Cr_10_, CrMnFeCoNi, and CrCoNi alloys are shown in Fig. 3a. Remarkably, all three alloys possess $\gamma _{{\mathrm{usf}}} \approx \gamma _{{\mathrm{utf}}}$ with small (or negative) $\gamma _{{\mathrm{isf}}}$. Similar IMPs were reported by several former theoretical woks^34,36^ (see also Supplementary Fig. 1). This particular shape of the *γ*-surface differs from that of conventional fcc alloys^27,31,42^ and has important implication on the deformation mechanism as explained in the following paragraphs. The *γ*-surfaces in Fig. 3a were obtained from alias shear (inset in Fig. 3a), i.e., by shifting the upper part of the fcc $\left\{ {111} \right\}$ layers along the $\langle 11\bar 2\rangle$ direction. In practice, this process requires a critical resolved shear stress, which raises a small but finite affine shear: all atomic planes are actually elastically strained along the shear direction (inset in Fig. 3b)^43^. The affine shear strain-dependence of $\left( {\bar \gamma _{{\mathrm{SF}}} - \bar \gamma _{{\mathrm{TW}}}} \right)$ is presented in Fig. 3b. Interestingly, increasing affine shear strain increases $\left( {\bar \gamma _{{\mathrm{SF}}} - \bar \gamma _{{\mathrm{TW}}}} \right)$ and thus favors twin nucleation for all alloys considered here. In particular, MTW is predicted in CrCoNi alloys as well with increasing affine shear strain. Therefore, in addition to the intrinsic materials properties, the strain conditions also sensitively affect the twinnability of these alloys. The sensitivity of the deformation mode to the shearing conditions should be attributed to the special shape of the *γ*-surfaces in Fig. 3a. We note that the beneficial effect of strain on the twinnability is mentioned in connection with the empirical deformation maps as well^22^.

For CrMnFeCoNi alloy, the measured critical twinning stress is around 235 ± 10 MPa^44^. Combining this value with the resolved shear modulus (48 GPa), for the affine shear strain we obtain ~0.8% as the characteristic value^45^. The Fe_40_Mn_40_Co_10_Cr_10_ and CrCoNi alloys are expected to possess slightly smaller and larger strains, respectively, as suggested by their EEBs (Fig. 3a). The impact of the strain on the temperature-dependent $\left( {\bar \gamma _{{\mathrm{SF}}} - \bar \gamma _{{\mathrm{TW}}}} \right)$ for the considered alloys is illustrated in Fig. 2 for 0.8 and 1.2% affine shear strains (open symbols). In the sheared Fe_40_Mn_40_Co_10_Cr_10_, transformation-induced plasticity happens only at very low temperature, otherwise MTW or regular TW is present. In CrCoNi, about 0.9% affine shear strain produces MTW at all temperatures, which is in line with the recent experiments^7^. In the sheared CrMnFeCoNi (strains ≳0.7%), MTW occurs for all temperatures below ~460 K, which explains the reported stress–strain behavior at cryogenic condition^6^. On the other hand, for CrMnFeCoNi, the calculation without shear strain predicts a martensitic transformation below ~300 K, which is consistent with the pressure-induced fcc–hcp phase transition^32,33^.

### Mechanism of metastable twinning

So far we defined MTW as twinning deformation when Δ*F* ≤ 0. Below, we reformulate the MTW condition using the SFE and related IMPs. An intrinsic stacking fault in an fcc lattice can be treated as a two-layer embryo with the hcp structure embedded in the fcc matrix. Accordingly, the SFE is often expressed as^46^ $\gamma _{{\mathrm{isf}}} = 2\Delta F/A + 2\sigma ^ \ast$, where *A* is the area of the stacking fault and σ^*^ is the pseudo-interfacial energy between the hcp embryo and the fcc matrix. Hence, $\gamma _{{\mathrm{isf}}} \le 2\sigma ^ \ast$ can be taken as the condition for the thermodynamic stability of the hcp lattice. Using our calculated $\gamma _{{\mathrm{isf}}}$ and Δ*F* values, for the present alloys we obtain σ^*^ ≈ 4–9 mJ m^−2^, which compares well with 8–9 mJ m^−2^ reported for Fe–Cr–Ni alloys encompassing about 14–20% Cr and 16–20% Ni^47^. In applications, σ^*^ is often considered to be independent of temperature^46,48^.

In terms of IMPs, twinning can occur if $\gamma _{{\mathrm{isf}}} \, > \, \gamma _{{\mathrm{utf}}} - \gamma _{{\mathrm{usf}}}$. Elemental fcc metals and austenitic steels^31,39,42,49^ have $\gamma _{{\mathrm{utf}}} \, {\mathrm{ > }} \, \gamma _{{\mathrm{usf}}}$, and thus a positive SFE is required to activate TW. The opposite case $\gamma _{{\mathrm{utf}}} \, {\mathrm{ < }} \, \gamma _{{\mathrm{usf}}}$ is characteristic to hcp metals^27,50^, for which however the large negative $\gamma _{{\mathrm{isf}}}$ forbids TW. These two cases provide the physical background for the phenomenological correlations between SFE and the deformation modes^22^. However, twinning can take place also when $\gamma _{{\mathrm{utf}}} \, \lesssim \, \gamma _{{\mathrm{usf}}}$ if $\gamma _{{\mathrm{isf}}}$ remains larger than a small (negative) critical value. Numerically, for twinning one should have $\gamma _{{\mathrm{isf}}} \,> \,2\delta$ where $\delta \equiv \gamma _{{\mathrm{utf}}} - \gamma _{{\mathrm{usf}}} - \gamma _{{\mathrm{isf}}}/2$ expresses the deviation relative to the so-called universal scaling law^51^. Theoretical calculations show that for fcc metals and alloys, *δ* can be both positive and negative^42^, and its absolute value is usually around a few mJ m^−2^. Combining this constraint for twinning with the one for the lattice instability, for the condition of MTW, we obtain $\delta \,< \,\gamma _{{\mathrm{isf}}}/2 \,< \,\sigma ^ \ast$. Hence, MTW can only be realized in systems where *δ* is below the pseudo-interfacial energy.

The above condition excludes from the MTW family, all metals and alloys, which possess $\gamma _{{\mathrm{isf}}} \,> \,2\sigma ^ \ast$ at all relevant temperatures. But for the present multi-component alloys^38^, as well as for the paramagnetic Fe^52^ or the low-Ni austenitic stainless steels^31^, $\gamma _{{\mathrm{isf}}}$ is negative at static conditions and increases with increasing temperature. In such systems, $\gamma _{{\mathrm{isf}}}/2$ usually scans the interval between *δ* and σ^*^ when changing the temperature, and thus the MTW mechanism might become effective within a specific temperature range. The sign of $(\delta - \sigma ^ \ast )$ in turn is decided by the chemistry and its magnitude is sensitive to the affine shear strain preceding the deformation. Since σ^*^ is usually small (typically below 10 mJ m^−2^), mostly alloys with very small or negative *δ* are expected to enter the MTW regime upon changing the temperature. Alloys with positive *δ* (but still below σ^*^), such as Fe–Cr–Ni^31^, are predicted to have a very narrow temperature interval for MTW. Finally, alloys with $\delta \,> \,\sigma ^ \ast$ transform directly from the normal TW region of the stable austenitic phase to the SF region with lowering the temperature.

**Fig. 4:**
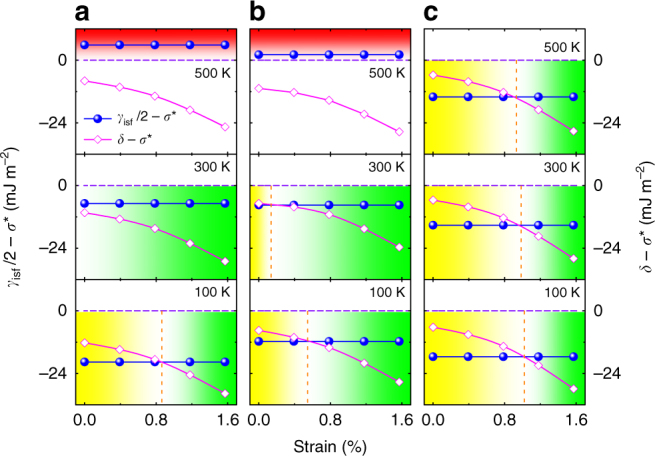
Twinning parameters as a function of the affine shear strain. **a**–**c** show the strain-dependence of ($\gamma _{{\mathrm{isf}}}/2 - \sigma ^ \ast$) and ($\delta - \sigma ^ \ast$) for three different temperatures for the Fe_40_Mn_40_Co_10_Cr_10_, CrMnFeCoNi, and CrCoNi alloys, respectively. Metastable twinning corresponds to $(\gamma _{{\mathrm{isf}}}/2 - \sigma ^ \ast ) > (\delta - \sigma ^ \ast )$ and $(\gamma _{{\mathrm{isf}}}/2 - \sigma ^ \ast ) \, < \, 0$ (green area), martensitic transformation to $(\gamma _{{\mathrm{isf}}}/2 - \sigma ^ \ast ) \, < \, (\delta - \sigma ^ \ast )$ (yellow area), and normal twinning to $(\gamma _{{\mathrm{isf}}}/2 - \sigma ^ \ast ) \, > \, 0$ (red area). The latter occurs in Fe_40_Mn_40_Co_10_Cr_10_ and CrMnFeCoNi alloys at high temperature but not in CrCoNi. Metastable twinning is present in all three alloys within certain strain–temperature windows

The two critical parameters for MTW, $(\gamma _{{\mathrm{isf}}}/2 - \sigma ^ \ast )$ and $(\delta - \sigma ^ \ast )$, are shown in Fig. 4 for Fe_40_Mn_40_Co_10_Cr_10_, CrMnFeCoNi, and CrCoNi as a function of affine shear strain and temperature. It is remarkable that all three alloys satisfy the $(\delta - \sigma ^ \ast )$ < 0 condition at all temperatures and strains from the figure and therefore they are prone to MTW. At the same time, the calculated relative SFEs $(\gamma _{{\mathrm{isf}}}/2 - \sigma ^ \ast )$ increase with temperature but remain negative below 410 K for Fe_40_Mn_40_Co_10_Cr_10_, 460 K for CrMnFeCoNi, and at all temperatures considered here in the case of CrCoNi. Hence, MTW should appear within certain temperature and strain intervals in all three multi-component alloys. These findings are in perfect line with the present theoretical predictions in Fig. 2 as well as with the available observations^3,6,7,20^.

### Stacking fault energy and metastable twinning

**Fig. 5:**
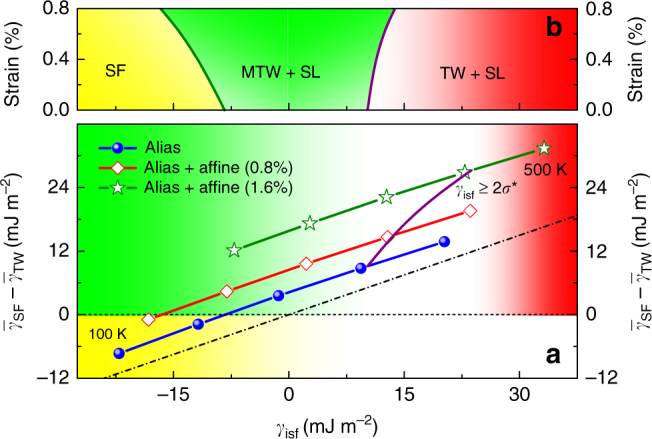
Deformation modes for the Fe_40_Mn_40_Co_10_Cr_10_ alloy as a function of intrinsic stacking fault energy ($\gamma _{\mathrm{isf}}$). Panel **a** shows the effective energy barrier difference for temperatures between 100 and 500 K, and for three different affine shear strains. The dash-dot lines marks the ideal relation $\gamma _{{\mathrm{utf}}} = \gamma _{{\mathrm{usf}}} + \gamma _{{\mathrm{isf}}}/2$ (i.e., the *δ* = 0 case). The solid line is the approximate boundary between metastable twinning (green area) and normal twinning (red area) regions in terms of $\gamma _{{\mathrm{isf}}}$. Martensitic transformation (yellow area) occurs for $\gamma _{{\mathrm{isf}}}$ < −8.4 mJ m^−2^ for the strain-free case, and this boundary is shifted towards lower values with increasing affine strain. The schematic deformation mode diagram shown in panel **b** is based on the theoretical data obtained for Fe_40_Mn_40_Co_10_Cr_10_

In order to express the deformation modes in terms of the experimentally accessible SFE, in Fig. 5a we plot the relationship between $\left( {\bar \gamma _{{\mathrm{SF}}} - \bar \gamma _{{\mathrm{TW}}}} \right)$ and $\gamma _{{\mathrm{isf}}}$ for Fe_40_Mn_40_Co_10_Cr_10_ for temperatures between 100 and 500 K and three affine strain levels. In the stress-free case, MTW is present for $\gamma _{{\mathrm{isf}}}$ between −8 and 10 mJ m^−2^, i.e., both negative and small positive SFE values are associated with TW. Increasing the affine shear strain, the maximum allowed $\gamma _{{\mathrm{isf}}}$ for MTW is shifted slightly towards positive values due to the weak stress-induced increase of σ^*^. Furthermore, the lowest SFE for which twinning is observed decreases strongly with strain and thus broadening the range for MTW. For 0.8% strain, which is close to the estimated value for CrMnFeCoNi, the lowest SFE where TW is the active deformation mode (in addition to SL) is −16.5 mJ m^−2^. The room-temperature SFE values for strains up to 0.8% vary between −1.3 and 2.3 mJ m^−2^, and lie well inside the MTW region. These SFEs are far below the empirical limit for twinning (10–16 mJ m^−2^) and confirm that classical SFE-based deformation models^22,23^ fail to account the observed twinning in this HEA^3^. In Fig. 5b, we propose a schematic deformation mode diagram based on the theoretical data obtained for Fe_40_Mn_40_Co_10_Cr_10_. We mention that the deformation maps for CrMnFeCoNi and CrCoNi (not shown) are qualitatively the same as that in Fig. 5b, but the boundaries between SF and MTW are slightly shifted towards larger SFE values. Like in the phenomenological diagram^22^, in Fig. 5b, the SF mode occurs for negative and the TW mode for positive SFE. Interestingly, the present theoretical SFE values for normal TW are above 7–14 mJ m^−2^ (depending on the affine shear level), which agree well with 6–12 mJ m^−2^ obtained by correcting the empirical critical values of twinning^24–26^ by the estimated strain contribution near the partial dislocations^53^. However, in the deformation mode map for MEAs and HEAs, there is a broad MTW region, which broadens towards both positive and negative SFE values with increasing affine strain. In contrast, in the case of traditional alloys like austenitic stainless steels, the MTW region is very narrow or completely missing.

The present theoretical exploration opens opportunities to fine tune the plastic flow mechanisms in HEAs. One important example is the enhanced twinning probability ($f_{{\mathrm{TW}}}$), which means that an increased number of randomly oriented grains exhibit twinning. In ideal metals (*δ* = 0), we obtain $f_{{\mathrm{TW}}}$ = 50% for $\gamma _{{\mathrm{isf}}}$ = 0 mJ m^−2^, meanwhile in metals with *δ* > 0, the twinning fraction turns below 50%. For instance, for Fe_68_Cr_20_Ni_12_ alloy^31^, *δ* = 7 mJ m^−2^ and $\gamma _{{\mathrm{usf}}}$ = 276 mJ m^−2^ give $f_{{\mathrm{TW}}}$ = 45%. However, in alloys exhibiting MTW, $f_{{\mathrm{TW}}}$ can reach values substantially higher than the previously established upper limit. For instance, the present results for Fe_40_Mn_40_Co_10_Cr_10_ yield *δ* = −4 mJ m^−2^ (neglecting the affine strain) and thus $f_{{\mathrm{TW}}}$ = 53%. Hence the TW fraction for the present HEA is ~18% larger than the value obtained for steels.

We have investigated the plastic deformation in multi-component alloys and presented a transparent atomic-level theory for the MTW mechanism. The theory is based on the EEBs derived from first-principle calculations. We focus on the high strength and high ductility alloys, Fe_80−*x*_Mn_*x*_Co_10_Cr_10_, CrMnFeCoNi, and CrCoNi, in which twinning was reported to be a fundamental deformation mechanism. Both theoretical and experimental results suggest that the studied alloys possess a thermodynamically unstable fcc lattice at ambient and cryogenic conditions, which questions the phenomenological plasticity models. In contrast to those models, the present theory demonstrates that twinning can be the primary deformation mode for metastable fcc alloys with a twinning fraction that surpasses the previously established upper limit. It is found that small affine shear strain can further amplify the MTW phenomenon. The disclosed behavior of the present MEAs and HEAs calls for a critical revision of the previously adopted design criteria regarding twinning-induced plasticity. Our quantum theory aided understanding of the plasticity shows that a precise control of the deformation mechanisms is feasible in these new families of multi-component alloys, which can ultimately facilitate the optimal harvesting of their properties.

## Methods

### Theoretical methodology

The present calculations were based on density-functional theory formulated within the generalized gradient approximation^54^ and the exact muffin–tin orbitals formalism^55^. The Kohn–Sham equations were solved within the scalar-relativistic and soft-core schemes. Since the Curie temperatures estimated via the mean field approximation^56^ are below 77 K, all alloys were described in the paramagnetic state modeled by the disordered local magnetic moment approximation^57^. The chemical and magnetic disorders were taken into account using the coherent potential approximation^55^. The free energies included the lattice expansion and magnetic entropy terms. For a randomly oriented polycrystalline alloy, the fraction of grains showing TW was estimated from the IMPs via^27^ $f_{{\mathrm{TW}}} = \frac{1}{{60^\circ }}\tan ^{ - 1}\left[ {\frac{{\sqrt 3 }}{3}\left( {1 - \frac{{4\delta }}{{\gamma _{{\mathrm{usf}}}}}} \right)} \right]$.

### Data availability

The data that support the findings of this study are available from the corresponding authors upon reasonable request.

## Electronic supplementary material

Supplementary Information
