# Supplementary material for: Twinning in metastable high-entropy alloys
Source: Nat Commun. 2018 Jun 18;9:2381. doi: 10.1038/s41467-018-04780-x (PMC6006257; doi:10.1038/s41467-018-04780-x)
Supplement: Supplementary file 1 — Supplementary Information [file 41467_2018_4780_MOESM1_ESM.pdf]

Supplementary Information

Twinning in metastable high-entropy alloys

Huang et al.

Supplementary Note 1

The intrinsic stacking fault energy $\gamma_{\rm isf}$ can be defined as $\gamma_{\rm isf} = (E_{\rm isf} - E_0)/A$, where $E_{\rm isf}$ and E_0 are the energies in the faulted and perfect lattices, A is the stacking fault area, respectively. The simplest way to obtain $\gamma_{\rm isf}$ is to make use of the axial interaction model (AIM)[1,2]. In the second and third order approximation, AIM gives $\gamma_{\rm isf}^{(2)} \approx 2(E_{\rm hcp} - E_{\rm fcc})/A$ and $\gamma_{\rm isf}^{(3)} \approx (E_{\rm hcp} + 2E_{\rm dhcp} - 3E_{\rm fcc})/A$, where $E_{\rm hcp}$, $E_{\rm dhcp}$, and $E_{\rm fcc}$ are the energies of the hcp, double hcp, and fcc structures, respectively. During the last years, people employed both supercell and AIM models to calculate $\gamma_{\rm isf}$ for multi-component alloys. Below we compare the present results for $\gamma_{\rm isf}$ with those obtained in former calculations.

Zhao et al.[3] studied the intrinsic stacking fault energy of CrMnFeCoNi and CrCoNi by using the special quasi-random structure (SQS) model. They found that the individual numerical values show a large scatter but the mean $\gamma_{\rm isf}$ values are negative for both alloys as shown in Supplementary Fig. 1. Furthermore, Zhang et al.[4] adopted the multiple randomly populated supercell method and reported that the mean value of $\gamma_{\rm isf}$ is -24 mJ m^{-2} at 0 K for CrCoNi, which turns out to be close to the present data (see Supplementary Fig. 1). Considering the differences in the underlying density-functional solvers (full-potential versus exact muffin-tin orbitals method), and how the chemical randomness (supercell versus coherent potential approximation) and magnetism (ferromagnetic versus disordered local magnetic moments) was treated, we conclude that the present and former results for the stacking fault energies are in good agreement with one another.

Ma et al.[5] investigated the temperature dependent free energy of the hcp and fcc structures of CrMnFeCoNi within the coherent potential approximation. Irrespective of the magnetic state, as shown in Supplementary Fig. 2, the hcp structure was predicted to be energetically favorable over the fcc structure at low temperatures. Li et al.[6] studied $Cr_{20}Mn_{20}Fe_{40-x}Co_{20}Ni_x$ (x = 0-20), and found that the hcp structure is stable at low temperatures and the finite-temperature contribution reduces the hcp stability. A similar phenomenon has been reported for the non-magnetic CrCoNi (see Supplementary Fig. 2)[4]. For the ferromagnetic CrCoNi, the hcp energy per atom is ~ 0.54 mRy smaller than the fcc one[4]. For the SQS described CoCrNi, Zhang et al.[7] found that the free energies of the hcp structure are generally lower than those of the fcc structure at low temperatures; Miao et al.[8] indicated that the energies per formula unit of the fcc and hcp structures are -1611.97 ± 0.26 mRy and -1613.88 ± 0.17 mRy, respectively. All these former theoretical predictions are fully consistent with the present results.

Supplementary Figures

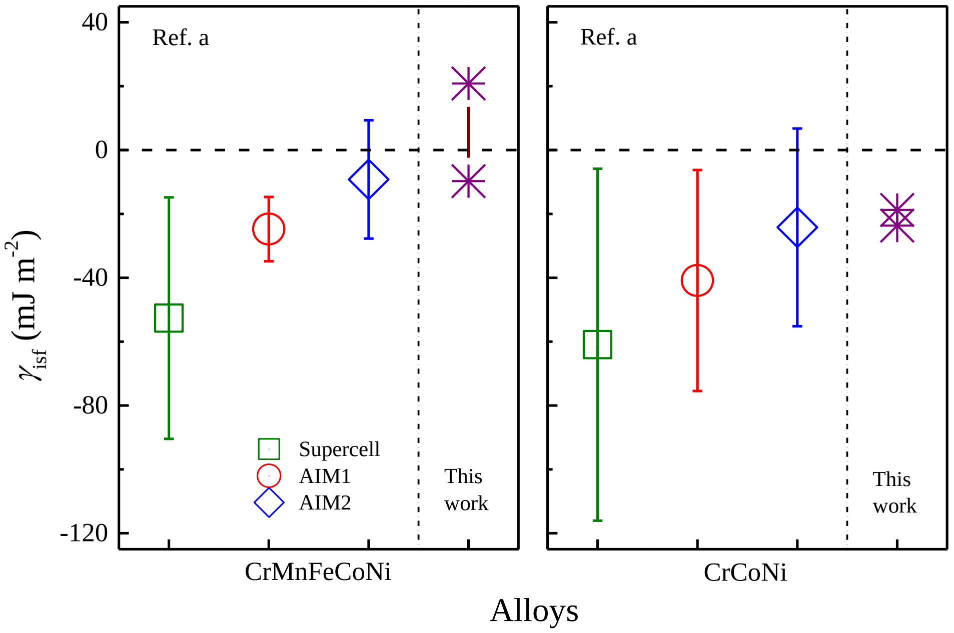

Supplementary Figure 1 | Intrinsic stacking fault energy γ_{isf} for the CrMnFeCoNi and CrCoNi alloys. The quoted theoretical data are from Ref. a [3] and correspond to the static conditions. AIM1 and AIM2 indicate results from the second and third order approximation of the axial interaction model, respectively. The corresponding data between 100 K and 500 K in this work are plotted for comparison.

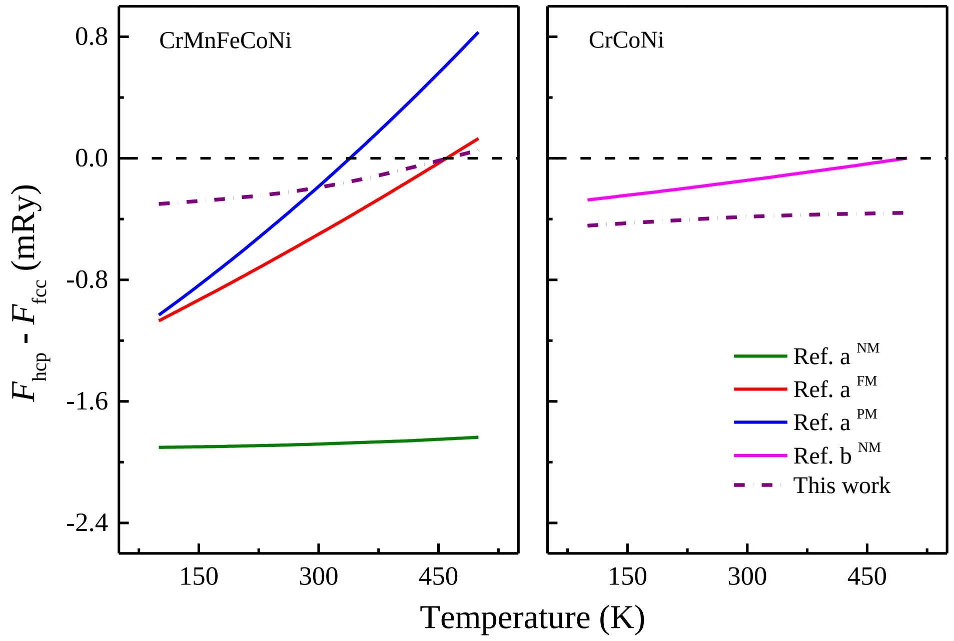

Supplementary Figure 2 | Temperature dependent structural energy difference ($F_{hcp} - F_{fcc}$) for the CrMnFeCoNi and CrCoNi alloys. The quoted theoretical data are from different magnetic configurations (NM: non-magnetic, FM: ferromagnetic, PM: paramagnetic) in Ref. a [5] and Ref. b [4]. The corresponding data between 100 K and 500 K in this work are plotted for comparison.

Supplementary References

1. Denteneer, P. J. H. & Haeringen, W. V. Stacking-fault energies in semiconductors from first-principles calculations. *J. Phys. C: Solid State Phys.* **20**, L883 (1987).
2. Cheng, C., Needs, R. J. & Heine, V. Inter-layer interactions and the origin of SiC polytypes. *J. Phys. C: Solid State Phys.* **21**, 1049 (1988).
3. Zhao, S., Stocks, G. M. & Zhang, Y. Stacking fault energies of face-centered cubic concentrated solid solution alloys. *Acta Mater.* **134**, 334-345 (2017).
4. Zhang, Z. *et al.* Dislocation mechanisms and 3D twin architectures generate exceptional strength-ductility-toughness combination in CrCoNi medium-entropy alloy. *Nat. Commun.* **8**, 14390 (2017).
5. Ma, D., Grabowski, B., Körmann, F., Neugebauer, J. & Raabe, D. Ab initio thermodynamics of the CoCrFeMnNi high entropy alloy: importance of entropy contributions beyond the configurational one. *Acta Mater.* **100**, 90-97 (2015).
6. Li, Z., Körmann, F., Grabowski, B., Neugebauer, J. & Raabe, D. Ab initio assisted design of quinary dual-phase high-entropy alloys with transformation-induced plasticity. *Acta Mater.* **136**, 262-270 (2017).
7. Zhang, F. X. *et al.* Pressure-induced fcc to hcp phase transition in Ni-based high entropy solid solution alloys. *Appl. Phys. Lett.* **110**, 011902 (2017).
8. Miao, J. *et al.* The evolution of the deformation substructure in a Ni-Co-Cr equiatomic solid solution alloy. *Acta Mater.* **132**, 35-48 (2017).
